# Echocardiographic Parameters in Patients with Pulmonary Arterial Hypertension: Correlations with Right Ventricular Ejection Fraction Derived from Cardiac Magnetic Resonance and Hemodynamics

**DOI:** 10.1371/journal.pone.0071276

**Published:** 2013-08-14

**Authors:** Tao Yang, Yu Liang, Yan Zhang, Qing Gu, Guo Chen, Xin-Hai Ni, Xiu-Zhang Lv, Zhi-Hong Liu, Chang-Ming Xiong, Jian-Guo He

**Affiliations:** 1 State Key Laboratory of Cardiovascular Disease, Fuwai Hospital, National Center for Cardiovascular Diseases, Chinese Academy of Medical Sciences and Peking Union Medical College, Beijing, China; 2 Department of Cardiology, Beijing Chaoyang Hospital Affiliated to Capital Medical University, Chaoyang District, Beijing, China; Vanderbilt University Medical Center, United States of America

## Abstract

**Background:**

Echocardiography is the most convenient method used to evaluate right ventricular function, and several echocardiographic parameters were studied in previous studies. But the value of these parameters to assess the right ventricular function in patients with pulmonary arterial hypertension (PAH) has not been well defined.

**Methods:**

Patients with PAH were observed prospectively. Right heart catheterization, echocardiography and cardiac magnetic resonance (CMR) were performed within 1 week interval. The correlations between echocardiographic parameters and right ventricular ejection fraction (RVEF) derived from CMR as well as hemodynamics were analyzed.

**Results:**

Thirty patients were enrolled including 24 with idiopathic PAH, 5 with PAH associated with connective tissue diseases and 1 with hereditary PAH. All echocardiographic parameters except right ventricular myocardial performance index (RVMPI) correlated significantly with RVEF (tricuspid annual plane systolic excursion [TAPSE], *r = *0.440, *P = *0.015; tricuspid annular systolic excursion velocity [S’], *r = *0.444, *P = *0.016; isovolumic acceleration [IVA], *r = *0.600, *P = *0.001; right ventricular fraction area change [RVFAC], *r = *0.416, *P* = 0.022; ratio of right ventricular transverse diameter to left ventricular transverse diameter [RVETD/LVETD], *r = *−0.649, *P<*0.001; RVMPI, *r = *−0.027, *P* = 0.888). After adjusted for mean right atrial pressure, mean pulmonary arterial pressure and pulmonary vascular resistance (PVR), only IVA and RVETD/LVETD could independently predict RVEF. Four echocardiographic parameters displayed significant correlations with PVR (TAPSE, *r = *−0.615, *P<*0.001; S’, *r = *−0.557, *P = *0.002; RVFAC, *r = *−0.454, *P = *0.012; RVETD/LVETD, *r = *0.543, *P* = 0.002).

**Conclusions:**

The echocardiographic parameters IVA and RVETD/LVETD can reflect RVEF independently regardless of hemodynamics in patients with PAH. In addition, TAPSE, S’, RVFAC and RVETD/LVETD can also reflect PVR in PAH patients.

## Introduction

Pulmonary arterial hypertension (PAH), caused by vascular remodeling of the small pulmonary arteries, is characterized by mean pulmonary arterial pressure (mPAP) ≥25 mmHg and pulmonary capillary wedge pressure (PCWP) ≤15 mmHg at rest assessed by right heart catheterization (RHC). [1] With pulmonary vascular resistance (PVR) increasing, the overloaded right ventricle (RV) changes morphologically and functionally, which impairs the capacity and survival of patients. [2] Recently, the role of the RV in PAH patients gains increasing attention from clinicians and researchers.

Pulmonary vascular remodeling and the RV dysfunction both play important roles in the pathophysiological process of PAH. Comprehensive evaluation of the condition of pulmonary hypertension (PH) patients requires the consideration of both the pulmonary circulation and RV function. RVEF that can reflect intrinsic RV contractility has been demonstrated to be an independent predictor of survival. [2]–[3] Cardiac magnetic resonance (CMR) imaging can accurately assess RV end-diastolic and end-systolic volume to calculate right ventricular ejection fraction (RVEF), [4], [5] which has become the reference standard technique for assessment of RV structure and function.

Echocardiography is the most common and convenient method used to evaluate RV function and hemodynamics of pulmonary circulation, and several echocardiographic parameters have been studied in previous studies, including RV fractional area changes (RVFAC), RV myocardial performance index (RVMPI), [6]–[8] tricuspid annular plane systolic excursion (TAPSE), tricuspid annular plane systolic velocity(S’) [9] and myocardial acceleration during isovolumic contraction (IVA). However, the value of these parameters to assess the severity and RV function in patients with PAH has not been well defined. Our previous study found the ratio of RV and LV end-diastolic diameter was a prognosis predictor in idiopathic pulmonary hypertension (IPAH) patients, [10] therefore we hypothesized it may also reflect the severity and RV function of patients with PAH.

Thus, in the present study, we attempted to analyze the correlations between the echocardiographic parameters and RVEF as well as hemodynamics, in order to systematically elucidate the role of these parameters in assessing the severity of PAH.

## Methods

### Patients Selection

PAH patients who were referred to Fuwai hospital between October 2010 and April 2012 for treatment were evaluated for this study. Inclusion criteria were mPAP≥25 mmHg and PCWP≤15 mmHg by RHC at rest. We excluded (1) patients with congenital heart diseases; (2) patients with unstable conditions; (3) patients with contraindication of CMR; (4) patients who refused to participate. Finally, thirty patients were recruited, and all of them underwent RHC, echocardiography and CMR within 1 week. The written informed consent was acquired from all patients enrolled. This study complied with the Declaration of Helsinki and was approved by the Institutional Review Board of Fuwai Hospital.

### Clinical and Hemodynamic Evaluation

Exercise capacity was evaluated by the WHO functional class (WHO-FC) and 6-minute walk distance (6MWD) according to the guidelines of the American Thoracic Society. [11] Hemodynamic parameters including mean right atrial pressure (mRAP), mPAP and PCWP were recorded in RHC examination. Pulmonary blood flow was measured by thermodilution method (the mean value of three-time measurement). PVR was calculated as the following equation: PVR = (mPAP-PCWP)/Pulmonary blood flow.

### Echocardiography

Echocardiography was performed by the Philips iE33 system, and images were analyzed offline after the procedure. TAPSE was acquired by M-mode image, and the cursor was placed through the lateral tricuspid annulus, the displacement of which from the end-diastole to the end-systole was measured. The S’ known as tricuspid annular systolic excursion velocity was measured by Tissue Doppler image (TDI) in the apical 4-chamber view. The RV isovolumic acceleration was calculated as the peak isovolumic myocardial velocity divided by the time to peak velocity measured by TDI at the lateral tricuspid annulus. RVFAC was calculated as follows: RVFAC = (RV diastolic area-RV systolic area)/RV diastolic area×100%. RV diastolic area and RV systolic area were obtained from the two-dimensional apical 4-chamber view. The RVMPI is the ratio of the isovolumic time and the ejecting time, which was measured in the same pulsed TDI. The isovolumic time was calculated by subtracting the ejecting time from the tricuspid closure time. The ratio of RV transverse diameter to LV transverse diameter was measured at the base in the end-diastole using the apical 4-chamber view.

### CMR

CMR was performed on a Siemens 1.5 T Sonata system (Siemens Medical Solutions, Erlangen, Germany), and phase offset errors was corrected by simultaneous ECG recording. Short-axis cine images including the whole LV and RV from apex to base were acquired by the gradient-echo pulse sequence (True-FISP by Siemens) (repetition time/echo time, 34 ms/1.6 ms; ﬂip angle, 60°; field of view, 280×340 mm^2^; matrix, 150×256 pixels; pixel size, 1.9×1.3; slice thickness, 6 mm; slice distance, 4 mm). The endocardium and epicardium outline of RV and LV were drawn manually using Argus software (Siemens Medical Solutions, Erlangen, Germany) by an experienced radiologist to obtain volumes of RV and LV.

### Statistical Analysis

Descriptive data for continuous variables with Gaussian distribution were presented as means ± standard deviation. Continuous variables were tested for adherence to a normal distribution with the Kolmogorov-Smirnov method. Pearson’s correlation coefficients were calculated to analysis the association of interesting parameters. Multivariate linear regression analysis was carried out to explain the role of echocardiographic parameters in predicting RVEF. Statistical significance was defined as *P*<0.05. Statistical analysis was conducted using SPSS version 16.0 for Windows (SPSS Inc., Chicago, Illinois).

## Results

Demographic, clinical and hemodynamic data of the thirty patients enrolled are shown in Table 1. The patients were mainly women (80%) and aged 30±10 years with the body mass index (BMI) 21.7±1.7 kg/m^2^. The subtypes of PAH were, idiopathic PAH 24 patients (80%); PAH associated with connective tissue diseases, 5 patients (16.7%); hereditary PAH, 1 patient (3.3%). The mPAP was 58±16 mmHg and PVR was 1168.5±509.4 dyn·s·cm^−5^. The cardiac index measured by thermodilution method was 2.39±0.98 L/min/m^2^. The data of echocardiography and CMR are presented in Table 2.

**Table 1:** Demographic, clinical and hemodynamic variables of patients with pulmonary arterial hypertension.

Characteristics	Value
Age, y	30±10
Gender	
Male, n(%)	6(20%)
Female, n(%)	24(80%)
Diagnosis	
Idiopathic pulmonary arterial hypertension	24(80%)
Pulmonary hypertension associated with connective tissue disease	5(16.7%)
Hereditary pulmonary arterial hypertension	1(3.3%)
Body mass index, kg/m^2^	21.6±1.6
Heart rate, beats/min	81±13
Blood pressure, mmHg	
Systolic	115±17
Diastolic	67±10
6 minute walk distance, m	380±104
WHO pulmonary hypertension function classes	
II, n(%)	19(47.5%)
III, n(%)	20(50%)
IV, n(%)	1(2.5%)
Mean right atrial pressure, mmHg	7±5
Mean pulmonary arterial pressure, mmHg	58±16
Pulmonary capillary wedge pressure, mmHg	9±5
Cardiac index, L/min/m^2^	2.39±0.98
Pulmonary vascular resistance, dyn·s·cm^−5^	1168.5±509.4
NT-pro brain natriuretic peptide, fmol/ml	1185.4±727.7

**Table 2:** Data of Echocardiography and Cardiac Magnetic Resonance.

Parameters	Value
Echocardiography	
TAPSE, mm	16.3±2.7
S’, cm/s	9.1±1.8
IVA, m/s^2^	2.2±0.8
RVFAC, %	28.9±7.0
RVMPI	0.77±0.18
RVETD/LVETD	1.45±0.47
Cardiac Magnetic Resonance	
RVEDV, ml	177.6±47.1
RVESV, ml	132.2±48.5
RVEF, %	27±12
LVEDV, ml	72.0±13.8
LVESV, ml	31.8±10.4
LVEF, %	55±9

TAPSE, tricuspid annual plane systolic excursion; S’, tricuspid annular systolic excursion velocity; IVA, right ventricular isovolumic acceleration; RVFAC, right ventricular fraction area change; RVMPI, right ventricular myocardial performance index; RVETD/LVETD, ratio of right ventricular transverse diameter to left ventricular transverse diameter; RVEDV, right ventricular end-diastolic volume; RVESV, right ventricular end-systolic volume; RVEF, right ventricular ejection fraction; LVEDV, left ventricular end-diastolic volume; LVESV, left ventricular end-systolic volume; LVEF, left ventricular ejection fraction.

### Correlations between Echocardiographic Parameters and CMR-derived RVEF

**Figure 1:**
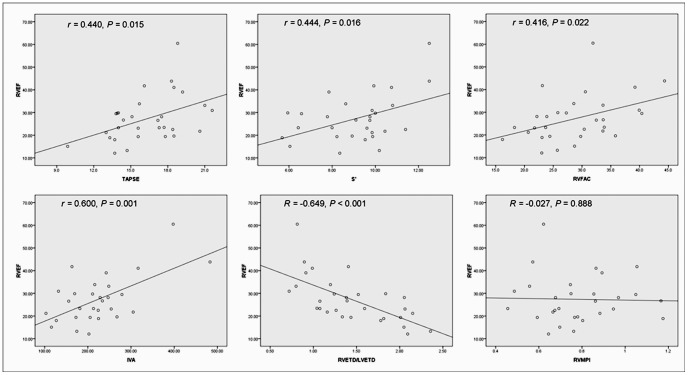
Correlations between echocardiographic parameters and right ventricular ejection fraction TAPSE, tricuspid annual plane systolic excursion; S’, tricuspid annular systolic excursion velocity; IVA, right ventricular isovolumic acceleration; RVFAC, right ventricular fraction area change; RVMPI, right ventricular myocardial performance index; RVETD/LVETD, ratio of right ventricular transverse diameter to left ventricular transverse diameter; RVEF, right ventricular ejection fraction.

Correlations between echocardiographic parameters and CMR-derived RVEF were shown in Figure 1. TAPSE, S’, RVFAC, IVA and RVETD/LVETD correlated significantly with RVEF (TAPSE, *r = *0.440, *P = *0.015; S’, *r = *0.444, *P = *0.016; RVFAC, *r = *0.416, *P = *0.022; IVA, *r = *0.600, *P = *0.001; RVETD/LVETD, *r = *−0.649, *P<*0.001). However, RVMPI had no significant correlation with RVEF (*r = *−0.027, *P* = 0.888).

### Correlations between Echocardiographic Parameters and Hemodynamic Parameters

Correlations between echocardiographic parameters and hemodynamic parameters are presented in Table 3. Four echocardiographic parameters displayed significant correlations with PVR (TAPSE, *r = *−0,615, *P<*0.001; S’, *r = *−0.557, *P = *0.002; RVFAC, *r = *−0.454, *P* = 0.012; RVETD/LVETD, *r = *0.543, *P* = 0.002). Moreover, TAPSE (*r = *−0.403, *P = *0.027), IVA (*r = *−0.408, *P = *0.025) and RVETD/LVETD (*r = *0.395, *P = *0.031) had significant relationships with mRAP. TAPSE, RVFAC and RVMPI showed significant correlations with mPAP(*r = *−0.378, *P = *0.039; *r = *−0.369, *P = *0.045 and *r = *0.367, *P = *0.046, respectively).

**Table 3:** Correlations between echocardiographic parameters and hemodynamics.

	PVR		mPAP		mRAP	
	*r*	*P*	*r*	*P*	*r*	*P*
TAPSE	−0.615	<0.001	−0.378	0.039	−0.403	0.027
S’	−0.557	0.002	−0.297	0.110	−0.294	0.114
IVA	−0.271	0.148	−0.027	0.886	−0.408	0.025
RVFAC	−0.454	0.012	−0.369	0.045	−0.297	0.110
RVMPI	0.210	0.265	0.367	0.046	−0.063	0.740
RVETD/LVETD	0.543	0.002	0.314	0.091	0.395	0.031

TAPSE, tricuspid annual plane systolic excursion; S’, tricuspid annular systolic excursion velocity; IVA, right ventricular isovolumic acceleration; RVFAC, right ventricular fraction area change; RVMPI, right ventricular myocardial performance index; RVETD/LVETD, ratio of right ventricular transverse diameter to left ventricular transverse diameter; PVR, pulmonary vascular resistance; mPAP, mean pulmonary arterial pressure; mRAP, mean right atrial pressure.

### Multivariate Regression Analysis for Predictors of RVEF

Multivariate linear regression analysis was used to investigate the associations between echocardiogaphic parameters and RVEF after adjusting for mRAP, mPAP and PVR. The results showed that only IVA and RVETD/LVETD could independently predict RVEF after the adjustment (*P* = 0.043 and *P* = 0.015) (Table 4). The model could account for 70.3% variation of RVEF.

**Table 4:** Multivariate regression analysis for predictors of right ventricular ejection fraction.

	Regression coincidence (95% CI)	*P*
mRAP	−0.464 (−1.158–0.229)	0.177
mPAP	0.065 (−0.358–0.488)	0.750
PVR	−0.005 (−0.021–0.012)	0.544
IVA	0.046 (0.002–0.090)	0.043
RVETD/LVETD	−13.636 (−24.313 - −2.960)	0.015
TAPSE	1.722 (−3.631–0.188)	0.074
RVFAC	−0.241 (−0.816–0.334)	0.391
S'	0.355 (−1.733–2.442)	0.726
RVMPI	−0.449 (−17.945–17.048)	0.958

mRAP, mean right atrial pressure; mPAP, mean pulmonary arterial pressure; PVR, pulmonary vascular resistance; IVA, right ventricular isovolumic acceleration; RVETD/LVETD, ratio of right ventricular transverse diameter to left ventricular transverse diameter; TAPSE, tricuspid annual plane systolic excursion; S’, tricuspid annular systolic excursion velocity; RVFAC, right ventricular fraction area change; RVMPI, right ventricular myocardial performance index.

## Discussion

Several echocardiographic parameters have been reported to assess the RV function and hemodynamics of pulmonary circulation. However, the value of these parameters remains controversial. In the present study, we used echocardiography and CMR to assess RV function and structure in PAH patients. We systemically analyzed the correlations between the echocardiograpic parameters and RV function as well as the hemodynamics, which may provide reference for comprehensively assessing the severity of PAH patients.

Despite several studies have validated the value of some echocardiographic parameters in assessing RV function, [12]–[14] which mainly focused on non-PH or healthy populations, relative little knowledge was present in PH patients. A recent study investigated the correlations between several echocardiographic parameters and RVEF measured by CMR, which enrolled 37 patients with PH of different etiologies (20 with PAH). [9] Different causes of PH may lead to diverse RV remodeling, [15] therefore, to reduce the bias, in the present study we only enrolled patients with PAH and excluded the patients with congenital heart disease. Moreover, we investigated six parameters including TAPSE, S’, RVFAC, IVA, MPI and RVETD/LVETD, and analyzed the value of these indices in predicting RVEF after adjusting for the hemodynamics.

Among the six echocardiographic parameters, TAPSE, S’, RVFAC, IVA and RVETD/LVETD correlated significantly with RVEF. However, after adjusting for the hemodynamics including mRAP, mPAP and PVR, multivariate regression analysis indicated that IVA and RVETD/LVETD were the only two independent predictors of RVEF. Unaffected by pre−/after-load, [16] IVA was reported to indicate early right ventricular dysfunction in obstructive sleep apnea and systemic sclerosis patients.[17]–[18] In the present study, we were the first to validate the accuracy of this parameter to evaluate the RV systolic function in PAH patients, and found it can reflect right ventricular function independently. Another parameter, the ratio of RVETD and LVETD is an index to reflect the remodeling of ventricles, which has been reported to be a predictor for prognosis of patients with IPAH [10] and acute pulmonary embolism. [19] In our study, RVETD/LVETD predicted RVEF independently and correlated significantly with PVR. Furthermore, it can be easily obtained in the 4-chamber view and less susceptible to the complicated RV geometry and indistinct endocardium, so this parameter may have potential to be a routine index to identify right ventricular dysfunction and disease severity in PAH patients.

Both TAPSE and S’ can reflect the longititudinal systolic function of the RV. TAPSE from M-mode echocardiograph has been reported to significantly correlate with RVEF derived from radionuclide angiography. [20]–[21] Recently, Sato *et al* reported that TAPSE and S’ (r = 0.86 and r = 0.63) significantly correlated with RVEF derived from CMR, [9] but the relationship was weaker in our study (r = 0.440 and r = 0.444). In the study by Sato *et al*, PVR of the patients were lower than that of the population in our study and the RV function of were relatively better (with RVEF of 38%±11% vs. 27%±12%). The differences may be due to the heterogeneity of patients included. With regard to the dilated RV in PH patients, circumferential contractility may also contribute a lot to RVEF. A CMR study by Kind *et al* reported that transverse wall movements provided important information of RV function in PH patients, in which TAPSE measured by CMR also had relatively weak relationship with RVEF (r = 0.458). [22] Since RVEF is determined by both the longitudinal and transverse systolic function, the TAPSE and S’ which can only reflect RV longitudinal systolic function may not be able to reflect RVEF perfectly. In addition, the significance of TAPSE and S’ may vary in patients with different severity.

Although the RVFAC is a common parameter for assessing RV systolic function, its two-dimensional measuring view can not completely reflect RVEF which is a three-dimensional parameter. Moreover, the hypertrophied RV trabeculae hinder us to distinguish the endocardium clearly in PH patients, therefore, accurate assessment of RVFAC can’t be assured. So the correlation coefficient in this study is moderate as well as the previous study, [9] indicating that RVFAC can only partially reflect RV systolic function.

The RVMPI has been reported to assess the combined systolic and diastolic function, [6]–[8] and significant correlation between the index and RVEF has been found in PH patients. [9], [23] However, in the work of Wang and his colleagues, significant correlation between RVMPI and RVEF by CMRI was not found in patients with ARVD and controls. [24] In the present study, we also failed to find a significant association between the two parameters. RVMPI is affected by the ventricular afterload [7] and the heterogeneity of population included existed in different studies, so different results turned up. Therefore, RVMPI may be not a stable parameter to assess RV systolic function.

With regards to the hemodynamic data, TAPSE, S’, RVFAC and RVETD/LVETD correlated with PVR significantly, which may provide some information for PH diagnosis and severity evaluation. In line with the previous study, RVMPI showed significant correlation with mPAP, but not PVR. [25] Moreover, there were no significant relationships between IVA and hemodynamics, which may indicate that this parameter is pre−/after-load independent. [26].

### Limitations

Firstly, echocardiography, CMR and RHC were not performed simultaneously, which may affect accurate comparisons of multiple methods. However, we excluded the patients with unstable conditions during the study period to reduce the bias. Secondly, the significance of imaging data may vary in different stages of PAH. The number of patients in the present study was small, which did not allow us to evaluate the data in patients with different stages.

### Conclusions

The echocardiographic parameters IVA and RVETD/LVETD can reflect RVEF independently regardless of hemodynamics in patients with PAH. In addition, TAPSE, S’, RVFAC and RVETD/LVETD can also reflect PVR in PAH patients.
